# An Analytic Hierarchy Process Contingency Analysis of Factors Affecting the Emigration Decision of Medical Doctors in Turkey

**DOI:** 10.3390/ejihpe14030041

**Published:** 2024-03-07

**Authors:** Cigdem Kadaifci, Y. Ilker Topcu, Enrique Mu

**Affiliations:** 1 Faculty of Management, Department of Industrial Engineering, Istanbul Technical University, Macka, 34367 Istanbul, Turkey; kadaifci@itu.edu.tr (C.K.); ilker.topcu@itu.edu.tr (Y.I.T.); 2 Department of Business Management, Accounting & Ethics, Carlow University, Pittsburgh, PA 15213, USA

**Keywords:** medical doctors, healthcare, healthcare sustainability, emigration, contingency analysis, analytic hierarchy process

## Abstract

While the importance of explicitly identifying and considering contingent factors such as decision content and context is widely accepted as a way to ensure the validity of the decision analysis for the specific task at hand, few studies include this. This research uses a contingency theoretical approach to study factors affecting the emigration decision of medical doctors (MDs) for the specific case of Turkey. The motivation for conducting this study arises from the observation that the growing trend in emigration among MDs from Turkey is having a significant impact on the country’s healthcare system. Dealing with the emigration of MDs is crucial for ensuring an effective and sustainable healthcare system, especially in terms of the availability of services, satisfaction, and employment of the healthcare staff. Contextual factors were explicitly identified through consultation with experts, while the generic factors were retrieved from the specialized medical migration literature. The Analytic Hierarchy Process method was utilized to prioritize the factors. Seventy-three participants were surveyed about their intention to either study or work abroad. The findings reveal that low remuneration and anxiety about their future due to the political situation in the country constitute the two most important factors driving the decision to emigrate.

## 1. Introduction

Medical doctor (MD) emigration is a global issue that involves doctors leaving their home country to practice medicine abroad. This phenomenon significantly affects the country of origin’s healthcare system as it has led to a shortage of qualified MDs and a strain on the resources that are available. A shortage of MDs can impede the delivery of efficient and high-quality healthcare services and results in challenging working conditions and an additional workload for non-emigrants in the hospitals of source countries (the ones from which doctors are emigrating) [1]. Moreover, the inexorably increasing number of emigrants among MDs leads to a significant impact on the sustainability of healthcare services in terms of both social and economic dimensions. Economic sustainability corresponds to “the practices that support long-term economic growth without compromising other dimensions of sustainability” and in the context of healthcare systems, it is mostly related to financial self-sufficiency, supporting research and innovation, and providing job opportunities, while social sustainability corresponds to “the ability of a healthcare system to enhance quality of life and improve well-being of a community” and among the healthcare-related measures, availability, employment, training, development and satisfaction of the staff can be listed [2]. Understanding the reasons for emigration may help to perceive the subtlest impact of them on sustainable practices.

India, the Philippines, Pakistan, Lebanon, Nigeria, South Africa, Ireland, Poland, and Romania are some of the countries where MD emigration is prevalent [3,4,5,6,7,8]. Turkey has been facing problems arising from the emigration of highly qualified individuals in recent years. Among those, the emigration of MDs deserves attention due to the increasing number of emigrants and its almost irreversible impact on the healthcare system left behind. Although the reasons have been discussed for a while by government and non-government organizations and the media in Turkey, a search in SCOPUS did not reveal any systematic study addressing this problem, with the exception of research by Genc [9] in The Lancet.

Before discussing the potential reasons for MD emigration, it would be beneficial to examine the numbers that provide proof of increasing emigration in Turkey. The Turkish Medical Association posted on their official twitter account that the number of MDs who have applied for the certificate of good standing, a document that allows MDs to practice abroad, increased from 59 in 2012 to 931 in 2020 and 2685 in 2022 [10]. Of these 2685 MDs who applied in 2022, 1344 were specialists.

There are several reasons why MDs choose to emigrate from Turkey. Among them, the challenging working conditions such as long working hours, inadequate resources and facilities are prominent. OECD data show that there were only 2 MDs per 1000 inhabitants in 2020, which ranks Turkey fourth from last among 37 countries [11]. The report also emphasizes that the number of doctors that represent “the doctors providing direct care to patients” lacks comparable data results in some countries including Turkey, which includes managerial or researcher positions in this number and which increases the total number of doctors by 10–15%. In addition to extreme workload, an increasing number of physical and emotional violent incidents perpetrated against MDs may have a significant impact on the emigration decision. When examining the literature, enactment of health policies, insufficiency of healthcare services, psychological factors associated with illness, conflicts between healthcare providers and patients, misunderstandings, dissatisfaction with medical care, feelings of neglect, extended waiting periods, illicit and improper requests, impact of alcohol or drug use, receiving distressing news, spread of misinformation through the media, and insufficient security measures were cited as the causes of violence against MDs [12,13,14]. The comparatively low salaries for MDs in Turkey make it difficult for them to provide for their families and fulfil their financial requirements. To be more specific, while the monthly poverty threshold is around TRY 23,000 (i.e., USD 1286, according to the indicative exchange rates on 29 July 2022 [15,16]), the base salary of a specialist is around TRY 18,000 (i.e., USD 1006) [17]. Other factors contributing to emigration are the lack of opportunities for career advancement and professional development due to a lack of funding and resources for research and innovation as well as political instability and uncertainty in the country.

All of these economic, social, and political factors lead to burnout and dissatisfaction of MDs and the decision of some to emigrate to another country. The goal of this study is to identify and prioritize these conflicting factors that lead MDs in Turkey to decide to emigrate abroad, using a multiple criteria decision making process.

As part of a recent effort to improve the validity of decision analysis, greater emphasis is being put on the consideration of contingent variables, namely decision content (e.g., strategic vs. non-strategic decisions) and decision context (e.g., decision-maker situation in terms of country, age, education) in the decision analysis process [18]. This approach, based on contingency theory, has been recognized as an important consideration in decision making [19]. In effect, while it could be tempting to think that the decision at hand (MDs deciding to emigrate) is an individual decision about leaving the country or not, the reality is that it is a significant decision made by a large group of important actors in the country. This study will identify the factors affecting this decision, both generic considerations (common to all doctors and countries) and those contingent upon the decision makers in the target country, through expert consultation. From a contingency theory perspective, the contingent factors in decision making are constituted by the decision type and its context [19]. According to Mintzberg’s classic definition [20], an MD’s decision to emigrate may be considered a strategic decision, given that it has important long-term consequences and will involve a large amount of resources for the decision maker. However, not all MDs are alike and each one may experience different contextual professional and personal situations that will influence their decision process. For this reason, this research includes medical specialists, specialty trainees and practitioners, as well as different age groups and genders. While it is expected that these sub-groups will have common ground in terms of the emigration decision factors, participants may differ strongly in their opinion of the relative importance of these factors depending on their situation. Finally, it is important to consider the decision context of Turkey. An MD deciding to emigrate out of Turkey may have very specific considerations that have not yet been discussed, to our knowledge, in the broad migration literature. For this reason, in this study we will solicit expert opinions to identify these contextual factors that have been apprehended through everyday experience.

The rest of the paper is organized as follows: the results of a literature review on the emigration of MDs are provided in Section 2. This section also offers significant inputs to the decision model in terms of generic factors. The theoretical framework of contingency theory is given in Section 3. The decision making framework is provided in Section 4. After the results and discussions are presented in Section 5, the conclusions and further research directions are given in Section 6.

## 2. Literature Review

**Figure 1:**
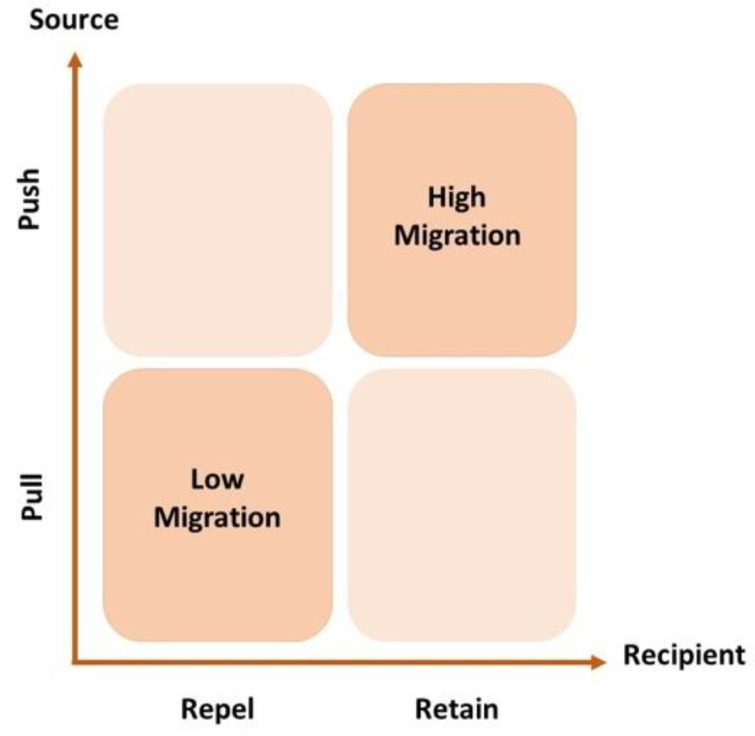
Conceptual framework for medical students’ decisions to train abroad. Source: Authors.

The extant literature on migration theory was used as the starting point to search for and identify known generic medical-migration factors. In effect, there has been a renewed interest in the migration phenomenon triggered in part by the significant migratory events in the summer 2015 [21]. Still, given the specific focus (medical doctors’ emigration), keywords such as “emigration” or “brain drain” combined with “doctors”, “physicians” or “healthcare staff”, as well as with “decision” or “decision making”, were used to access relevant articles in the SCOPUS database. Akl et al. was a particularly useful resource [3], as they used a framework of analysis that considered the presence of push or repel factors in the source country (e.g., low salaries, lack of professional opportunities) that drive the MD to emigrate as well as pull or retain factors (e.g., high salaries, need of professionals) that attract MD immigrants into the destination country. More recently, this model has been revised to distinguish between migration factors—conditions that may shape migration—and migration drivers as activated factors which, together, create the structural conditions within which people make decisions about whether to move or stay put [22]. For this reason, it is helpful to explore the migration factors in terms of this push/pull action in the source/destination countries. Based on [3], the proposed push/pull framework of the migration factors, a graphical conceptual model for the discussion of these factors is proposed in Figure 1. This constitutes the conceptual framework for the discussion of the extant literature on known MD generic migration factors.

The phenomenon of brain drain from developing countries, particularly in the medical sector, has become a critical issue worldwide. Domagała et al. [23] conducted a qualitative study to investigate the emigration experiences of Polish health professionals, including the reasons for leaving, challenges faced, and the effects of emigration on the Polish healthcare system. The study found that the primary drivers of emigration were inadequate salaries, suboptimal working conditions, and a lack of professional growth opportunities in Poland. The authors also identified factors that attracted Polish healthcare professionals to other countries, such as better compensation, a higher standard of living, and access to more advanced medical technologies. The study underscored the negative impact of emigration on the Polish healthcare system, including a shortage of medical staff and a reduction in the quality of care.

In 2020, a study of the Romanian medical diaspora revealed that low salaries, poor working conditions, and limited career opportunities were the primary factors that motivated MDs to migrate from Romania to Western European countries with strong pull factors such as better working conditions and higher salaries [4]. The authors also discussed the negative effects of brain drain, such as a shortage of medical staff and increased strain on Romania’s healthcare system.

Gouda et al. [5] conducted a study in Ireland to explore the migration intentions of Irish medical students and the factors that influenced their decision to migrate. The authors identified push factors such as the economic recession in Ireland, the associated lack of employment opportunities, and the perceived poor quality of the Irish health system as significant factors influencing the migration intentions of Irish medical students [5]. The study also highlighted the potential negative impact of medical brain drain on the Irish health system, including a shortage of medical staff and reduced quality of care. Four years later, another study investigated the reasons for doctor emigration from Ireland, with a specific focus on the role of declining job quality [6]. The study found that poor working conditions, low pay, long working hours, and limited opportunities for career progression contributed to a decline in job quality for doctors in Ireland. Consequently, many doctors opted to emigrate to countries such as the UK, Canada, and Australia, where there were pull factors such as better working conditions, higher salaries, and more opportunities for professional development. The authors emphasized that doctor emigration had negative effects on the Irish health system, including a shortage of medical staff and increased pressure on the remaining doctors.

Nigeria is one of the countries strongly affected by MD emigration. A research study in 2022 examined the reasons behind physician emigration from Nigeria and the implications for the Nigerian health system [7]. The study identified factors such as poor working conditions, low salaries, and a lack of opportunities for professional development as major drivers of physician emigration. The authors also identified push factors, such as political instability, insecurity, and poor living conditions, which forced physicians to emigrate. In another study that same year, the authors examined the impact of the COVID-19 pandemic on health-workforce brain drain in Nigeria [24]. The study revealed that the pandemic made existing challenges in the Nigerian health system worse, including inadequate funding, poor working conditions, and low remuneration, leading to increased emigration of health workers. The authors identified various push factors, such as high workload, exposure to infectious diseases, and inadequate protective equipment, as well as pull factors, such as better salaries and working conditions, and career opportunities in other countries.

A research team in Pakistan investigated the reasons why medical students in the country decide to pursue their careers abroad, and identified various factors that contribute to brain drain, including economic, educational, political, social, and professional considerations [8]. The authors classified these factors as push and pull factors from donor and recipient countries, respectively. The study emphasized the necessity for targeted policies and initiatives to address these factors. Consequently, the study’s findings provide crucial insights into physician migration and can significantly contribute to developing policies aimed at retaining medical students in their home countries.

The factors causing graduating Lebanese medical students to migrate were investigated by [3]. The study revealed that the main reasons for migration (push factors) were the unstable political situation, economic difficulties, and a lack of career opportunities in Lebanon. Moreover, the authors discovered that a significant proportion of medical students who planned to leave had a desire to come back to their home country in the future, but only if the country’s conditions improved. The authors concluded that the decision to migrate was multifaceted and was influenced by both individual and contextual factors.

A more geographically encompassing study examined the migration of healthcare workers from developing countries and suggested practical strategies to manage this trend [25]. The authors identified various push factors that influenced the migration of healthcare workers, including insufficient salaries, substandard working conditions, and limited career prospects in developing countries. Conversely, better salaries, improved living standards, and more advanced training opportunities in developed countries acted as pull factors for healthcare workers from developing countries.

For a proper study of healthcare migration patterns and their impact on the health systems of both source and destination countries, Diallo [26] emphasized the significance of enhancing data collection and reporting systems in order to gain a better comprehension of healthcare-worker migration patterns and their impact on the health systems of both source and destination countries. The author recommended the adoption of standardized definitions and terminology to simplify international comparisons and enhance the precision of migration estimates. The study highlighted the necessity of a collaborative strategy to tackle the obstacles associated with measuring healthcare worker migration and its consequences on global health.

In summary, the push/pull factors that drive the emigration of medical professionals have been identified and studied in countries like Poland, Romania, Ireland, Nigeria, Pakistan and developing countries in general. The main driving (push) factors for emigration, although not unique, are low salaries, poor working conditions and limited career opportunities. When the potential destination country has the proper (pull) attraction factors such high salaries, good working conditions and professional opportunities, the conditions are created for a high rate of migration among healthcare personnel. Also, Ref. [3] found that the migration decision is multifaceted and influenced by individual and contextual factors.

## 3. Theoretical Framework

The literature review, using Akl et al.’s push/pull migration theoretical framework [3], allowed the identification and discussion of many generic factors (i.e., related to most countries) driving the emigration of medical professionals. However, these studies are only partially helpful for the proposed objectives of the present study, which aims to answer the following research questions:

1. What are the driving factors for MDs’ emigration in Turkey?
2. What is the importance of the different factors driving MDs’ decision to emigrate in Turkey?

To answer the first question, it is necessary to identify the generic factors provided by the extant literature, and then to identify the contingent factors for the target decision makers in Turkey. A contingency theoretical approach requires the interview of experts that can provide new perspectives and identify contextual factors related to the decision factors [27].

**Figure 2:**
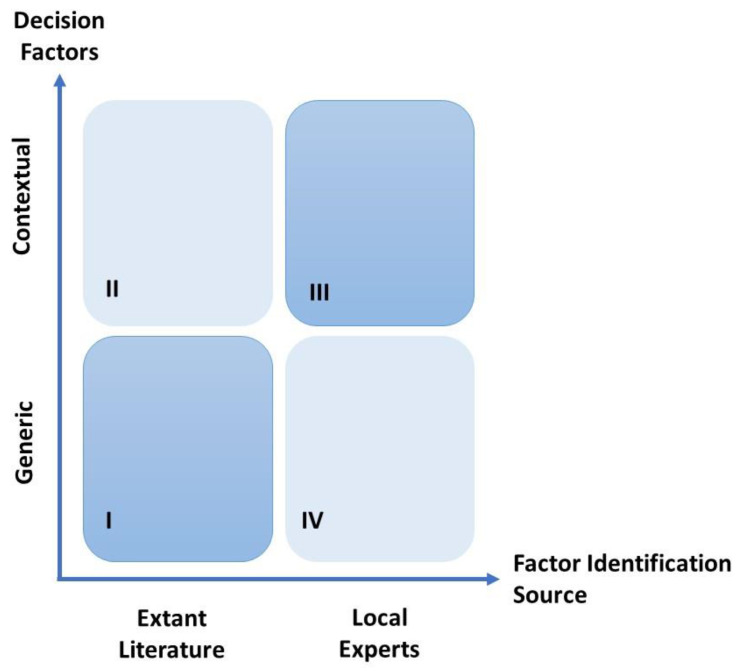
Matrix of decision factors versus factor identification. Source: Authors.

As previously stated, the overall approach will require the identification of medical migratory-decision generic factors available in the extant literature, as well as the contextual factors provided by experts, as shown in Figure 2.

While some contextual factors may be available in the literature if experts have published their specific knowledge (quadrant II), the extant literature review is focused on identifying the factors at large as seen by the discipline without consideration of the time and place (quadrant I). Similarly, experts are more qualified to provide relevant contextual factors related to the specific situation (quadrant III), but may also complement the identification of factors that are available in the literature (quadrant IV).

To answer the second question, which aims to reveal the importance of the factors influencing the emigration decision, a multiple criteria decision making framework is necessary, as this decision is multifaceted and influenced by various individual and contextual factors.

Before continuing, it is worth mentioning the rationale for the use of the Analytic Hierarchy Process (AHP) as the decision factor prioritization method in the present study. The AHP is a method of prioritizing decision factors using a pairwise comparison judgment approach to factors to create a pairwise comparison matrix and derive the priorities based on the calculation of the matrix eigenvector [28]. While the method is not without detractors, it has become theoretically robust over the years, and because it combines simplicity and rigor it is particularly useful in situations that involve a broad spectrum of stakeholder participation in the decision process [29].

Participants are not required to understand the details of the AHP methodology to use it, since all they need to provide is their judgment on the relative importance of one of two decision factors being compared at any given time. This has greatly favored the use of the AHP in decision analyses surveying the opinions of the actual stakeholders. This is very desirable in the analysis of social problems such as MDs’ migration decisions, since it has allowed the participation of a large number of MDs in the present study. Readers are referred to the original work by [28,30] or more recent introductions such as [31] for the specifics of the method.

In 1996, Saaty proposed the Analytic Network Process (ANP), which is an AHP generalization for the case of decision-element interdependencies, outer-dependencies and feedback [32]. In the context of the present study, it would refer to possible inter-correlations among the emigration factors. However, once the decision factors were identified, it became clear that any correlation among them was rather spurious, as can be concluded from the definitions of the decision model elements in Section 4.1. For this reason, a decision was made to use the AHP for the research at hand, given that it has the added advantage of requiring far fewer pairwise comparison questions than the ANP, decreasing the cognitive load and valuable time required of the MD participants.

## 4. Decision Making Framework

**Figure 3:**
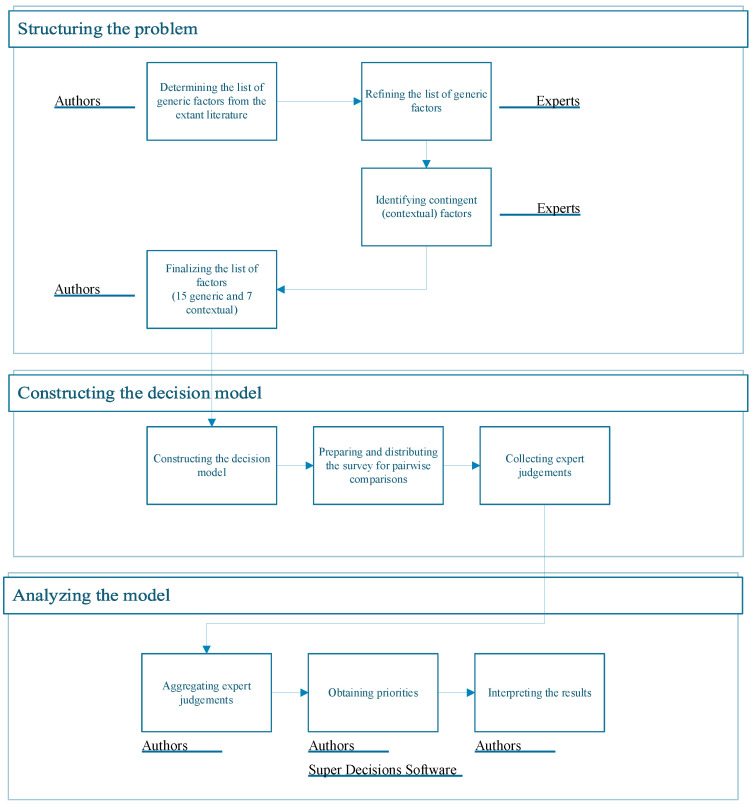
The flowchart of the methodology.

This study proposes a multiple criteria decision making (MCDM) process to determine the relative importance of the factors driving the emigration decision of MDs in Turkey. The MCDM process followed in this study consists of three stages, as can be seen in Figure 3: (i) structuring the problem, (ii) constructing the decision model, and (iii) analyzing the model.

### 4.1. Structuring the Problem

**Figure 4:**
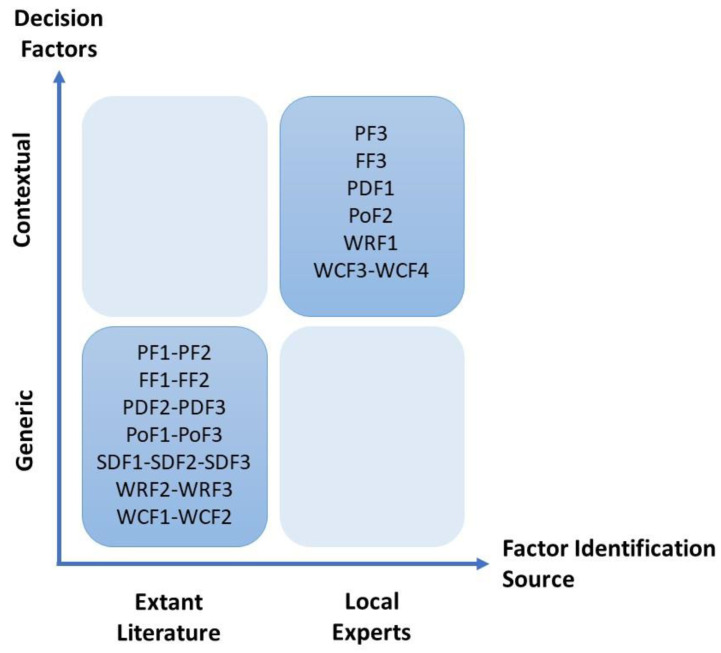
Matrix of generic and contingent (contextual) factors. Source: Authors.

First, the generic factors for the emigration decision were determined, based on a detailed literature review. A list of 32 factors was sent to three MDs, one of whom is a medical practitioner with eight years of experience and the other two full-time researchers in medical schools. They reviewed the list to properly define the factors and eliminate redundancy. The experts were also asked to add any country-specific contingent (local) factors that were particular for the context of Turkey, following similar practices in this type of contingency approach to the decision making process [27]. Through this process, 22 factors were classified into seven groups, as shown in Table 1 and Figure 4.

Psychological Factors (PFs) refers to the concerns and perceptions of MDs regarding their profession.

Recent studies demonstrate that the lack of appreciation from society and the government [33] and the expectation of better recognition [8] are among the factors causing MDs, especially in underdeveloped or developing countries, to emigrate. Accordingly, “perceptions regarding the loss of professional reputation” (PF1) was included in the model.

In addition, according to the annual reports of the Turkish Ministry of Health, the number of healthcare staff who used a “white code”—an emergency response for a violent person–increased from 7751 in 2017 to 101,984 in 2021 [34,35]. Violence and harassment against healthcare staff are being examined under the concept of workplace violence, which is classified as psychological and physical violence [36]. The physical violence includes “beating, kicking, slapping, stabbing, shooting, pushing, biting and pinching”, while psychological violence includes “verbal abuse, bullying/mobbing, harassment and threats” that is perpetrated by patients, members of organizations in the healthcare industry, peers, or superiors [37]. Workplace violence has been found to be significantly associated with anxiety and depression, which eventually leads to psychological depletion [36]. Hence, despite being classified as a political factor by [3], “personal security concerns” (PF2) was considered as a psychological factor in the case of Turkey due to the dramatic increase in patient-related violence and harassment incidents against healthcare staff.

As the three MDs indicated, “concerns about ethical violations by colleagues” (PF3) has originated from the tremendous increase in the number of medical schools in Turkey. As of February 2023, there are more than 120 medical schools (70% public, and 30% foundation universities) with almost 18,000 graduates per year [38]. The MDs emphasized that pioneer medical schools such as İstanbul, Hacettepe, and Ege each have their own training approach, which has an impact on both the diagnosis and treatment methodology of the trainees. The increase in the number of medical facilities undermines these traditional training approaches and causes significant concern regarding the quality of medical training, and thereby the qualifications of the graduates. For all these reasons, “concerns about ethical violations by colleagues” (PF3) constitutes a contingent factor in the case of Turkey.

Financial Factors (FFs) are the economic living standards of MDs. As previously mentioned, the base salary of MDs in Turkey is below the monthly poverty threshold. Considering that remuneration is a factor commonly addressed by researchers as an important reason for emigration [3,4,5,6,7,8,23,24,25,26], “low remuneration” (FF1) was included in the model. In Turkey, a performance-based salary system consisting of base and incentive payments is applied for healthcare staff [39]. The goal of this salary system is to improve the healthcare services and increase their efficiency and quality by ensuring that the remuneration is proportional to the quality of care provided. Accordingly, the incentive pay includes items such as general and specialized operations during or outside of working hours, overtime, reported patient satisfaction, etc. As is the case in most underdeveloped and developing countries, “inadequate performance payments” (FF2) are present in Turkey. However, MDs also emphasize that the incentive calculations are not fair and constitute a contingent factor for the country due to the following reasons: (i) they are not equally applied to each type of institution, (ii) incentives force the healthcare staff to treat more patients per day (e.g., for some specialties in public universities MDs have to treat one patient every five minutes), and (iii) the payments for MDs who are suffering from serious diseases and have a medical report for longer than a certain period are cut. For these reasons, the contingent factor “unfair performance payments” (FF3) has been included among the financial factors to consider.

Personal Development Factors (PDFs) are related to research and development activities gained through projects or scientific conferences, training, and other experiences. “Lack of training and self-development” (PDF2) [3,4,5,26] and “lack of experience opportunities” (PDF3) [5,25] are addressed in the extant literature, but “lack of research funds” (PDF1) provided by the government and healthcare authorities in Turkey was noted by MDs as inhibiting their development, and was considered rather contingent on the circumstances in the country.

Political Factors (PoFs) cover the feeling of “frustration with the political situation” (PoF1) and concerns about “political ethics and transparency issues” (PoF3) [3]. In addition, MDs indicated that they feel anxiety about the future of their profession due to the political atmosphere in Turkey (PoF2), which hence constitutes a contingent factor in this study.

Similar to other underdeveloped and developing countries, System Design Factors (SDFs) leading to emigration decisions are constituted by “dysfunctional healthcare systems” (SDF1), “poor professional standards” (SDF2), and “unfair competition for the promotion and managerial positions” (SDF3) [3].

For Working Relationship Factors (WRFs), “unsatisfactory mentorship” (WRF2) and “distressing relationships with peers or other healthcare staff” (WRF3) [3] are considered. Bullying/mobbing could also be considered as a psychological factor when the violence and harassment are perpetrated by patients. However, in our case, peer or superior bullying (WRF1) is classified as a working relationship contingent-decision factor.

In *Working Conditions Factors (WCFs)*, there are two generic and two country-specific factors. In addition to the “long working hours” (WCF1) [3,8] and “lack of equipment and personnel” (WCF2) [3,4] that MDs are suffering from in their practices, “compulsory service liability” (WCF3) and “assignment of doctors against their will” (WCF4) are considered in this group. The latter two factors are specific to Turkey. The Health Services Basic Law (No:3359-additional article:3) imposes a 300–600 day compulsory service on the MDs who are newly graduated from medical school or complete their specialty training, depending on the socio-economic development ranking of the region [40]. To clarify the compulsory service process, it is necessary to know that there are six different zones in the country and MDs who are responsible for compulsory service indicate a certain number of preferences. The open positions in a particular hospital are randomly chosen from among those who preferred that hospital. Although students know about the compulsory service when choosing their profession (or professional education area), doctors still consider this a factor among the reasons for emigration.

A summary of the decision factor groups and factors and their corresponding generic or specific (contingent) type is shown in Table 1.

### 4.2. Constructing the Decision Model

**Figure 5:**
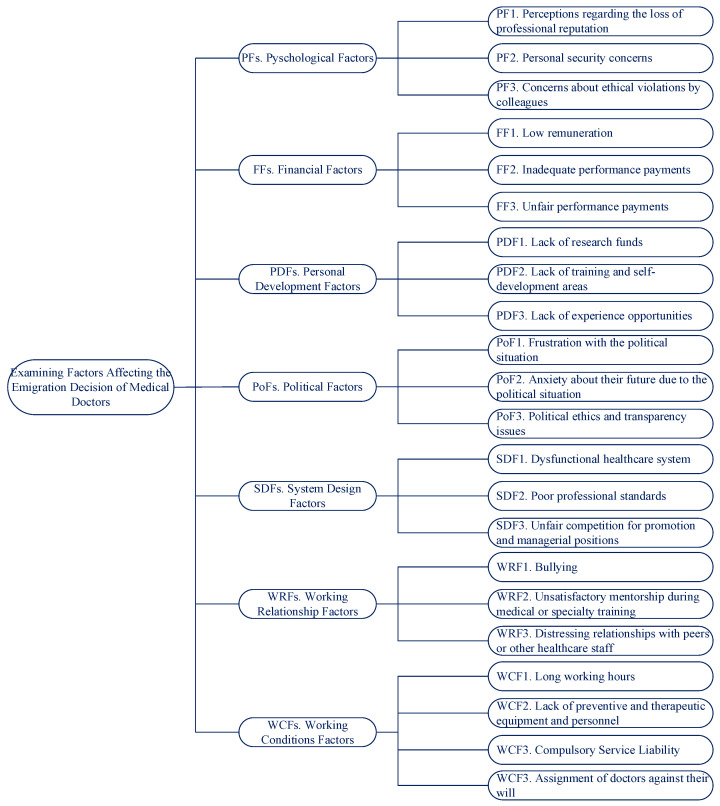
The decision model.

After determining the decision factor groups and factors, the decision model in the form of a hierarchy was constructed as given in Figure 5. An online survey questionnaire was prepared [41] to examine which of the factor groups and factors are of greater importance on the decision to emigrate of MDs in Turkey. The questionnaire (in Appendix A) included 45 pairwise comparisons and seven demographic questions in addition to four questions specific to MDs who have already emigrated. The questionnaire was administered to a sample of medical participants, who responded anonymously.

Participants were invited to participate via professional associations and social media and 127 of those who responded were eligible (i.e., MDs or medical students) to participate. Five of the eligible participants eventually declined to participate in the survey and forty-four of those who agreed were not included in the analysis due to partially assessing the pairwise comparisons and/or not being consistent in their assessments. An acceptable consistency ratio is 10%, as Saaty indicated; for more details, the readers may refer to [42]. Consequently, 73 participants provided usable survey answers. Based on this, the survey response rate was 57.4%, which is very high for this type of study.

The demographic information of the 73 participants shown in Table 2 demonstrates that 56.16% of the participants are female. The differences in age, ranging from 18 to over 50, may provide a comparison of preferences among different groups.

More than half of the participants (58.91%) graduated after 2010 and 54.8% of the MDs are either specialists or continuing their specialty training. In Table 3, the specialty areas of the MDs are provided; the 25 different specialty areas also allow for different perspectives in the analysis.

In addition to characteristics that enable the analysts to observe the preferences of different demographic groups, the survey also asked about emigration intention. Based on the European Commission’s definition of emigration, which states “*In the global context, the act of departing or exiting from one state with the intention to remain abroad for a period exceeding one year*” [43], this study considers participants who want to work or study abroad to have an intention to emigrate, regardless of their desire to settle elsewhere permanently. This approach has theoretical support in the work of Carling [44], recently revised by Carling J. and Schewel K. [45], who proposed a general aspiration/ability framework for the study of international migration. Migration aspiration is defined as the conviction that to migrate is preferable to non-migration. Still, for migration to occur, the ability to do so is needed (e.g., the skills and opportunity). It is argued that the intention to work or study abroad for more than a year not only constitutes emigration, technically speaking, and has great potential to provide the aspiration (e.g., due to better salaries, training and overall job opportunities), but more importantly, provides the ability to do so (e.g., due to established professional contacts and overall socialization). In summary, the suitability of the approach used to study medical emigration intention in the current study is supported by both EU definition and the migration aspiration/ability theoretical framework.

While 49.32% of the participants either want or definitely want to work or study abroad, 23.29% have not decided yet. Furthermore, out of the three participants who are currently abroad, two are in Europe (Germany and Switzerland) and one is in the USA. When we examined the return intention of those who are currently abroad, two indicated that they will not return, while one has not decided yet. This also suggests that it is very reasonable to include all those who state that they plan to study abroad in the intention-to-emigrate group. Since the return intention has commonly been examined for the people who already emigrated [46,47,48,49], the return intention of those who are currently in Turkey was not investigated in this study.

### 4.3. Analyzing the Model

In the final stage, the judgments of the participants were aggregated following AHP standard practices for group decision making and the relative importance of the migration factors were obtained [42]. The analyses were performed using Super Decisions v3.2 software [50], which was provided by the Creative Decisions Foundation [51]. The geometric means of the pairwise comparison assessments were calculated and entered into the software and the inconsistencies were checked before running the model. The highest inconsistency ratio for the overall assessments was 0.57%. The list of inconsistencies with respect to the goal and factor groups, both for the overall assessments and for the demographic groups, is given in Appendix B, Table A1, which shows that no inconsistency was above 10%.

## 5. Results and Discussion

**Figure 6:**
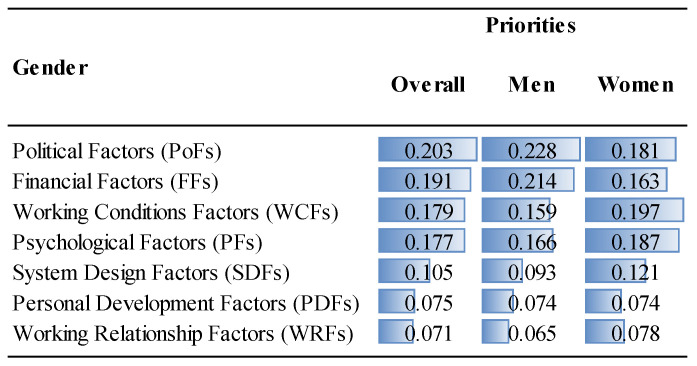
The priorities of the factor groups (Overall vs. Gender).

**Figure 7:**
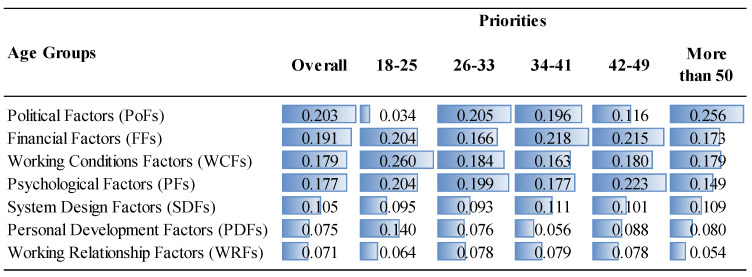
The priorities of the factor groups (Overall vs. Age Groups).

**Figure 8:**
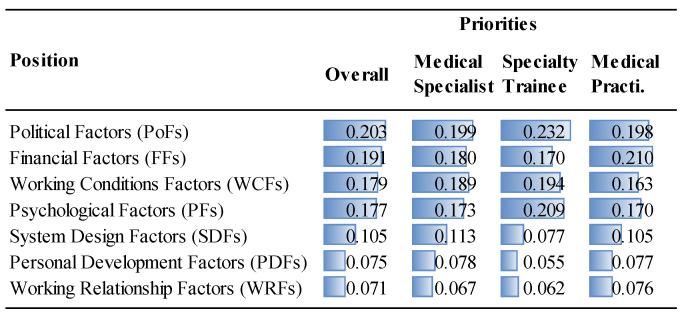
The priorities of the factor groups (Overall vs. Position).

**Figure 9:**
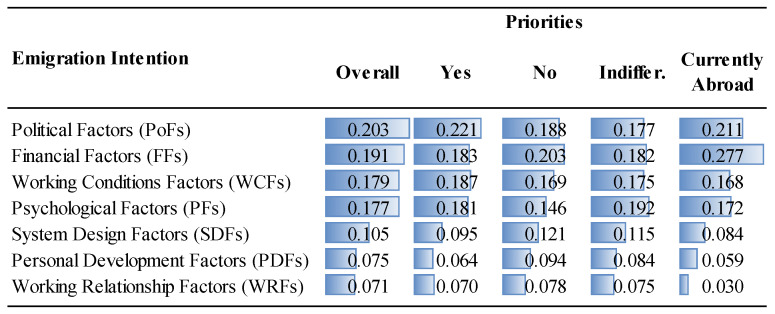
The priorities of the factor groups (Overall vs. Emigration Intention).

**Figure 10:**
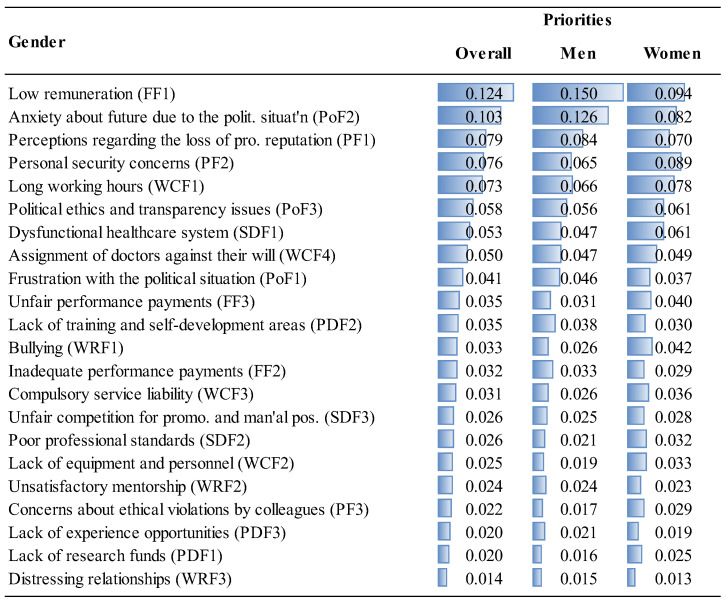
The priorities of factors (Overall vs. Gender).

**Figure 11:**
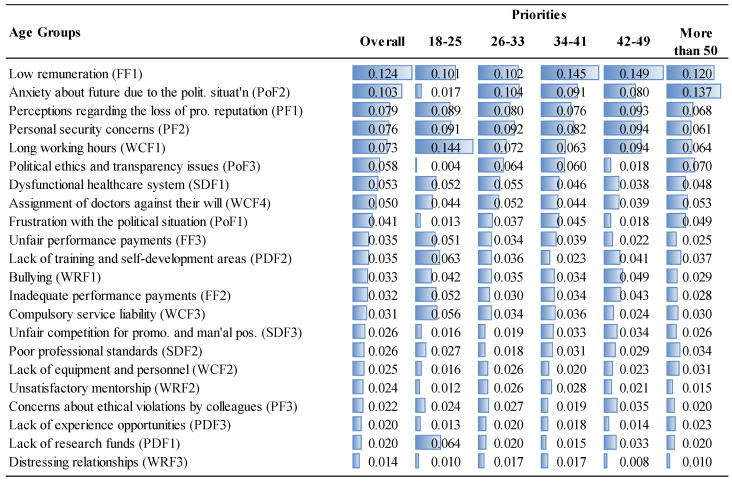
The priorities of factors (Overall vs. Age Groups).

**Figure 12:**
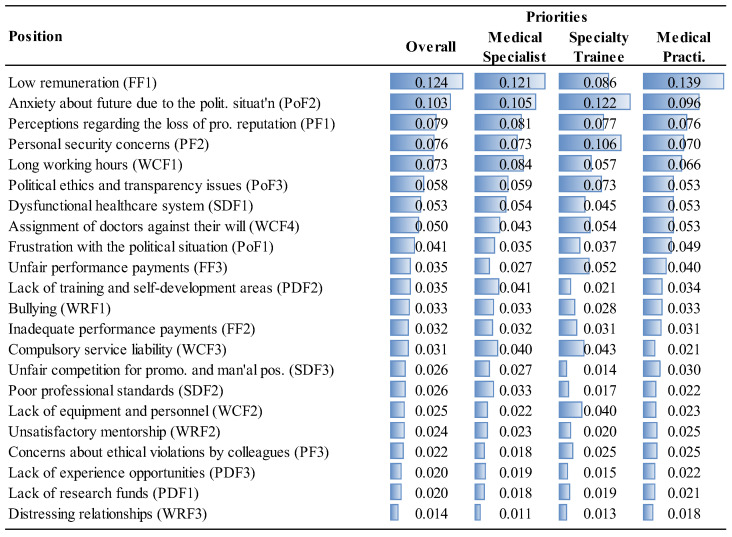
The priorities of factors (Overall vs. Position).

**Figure 13:**
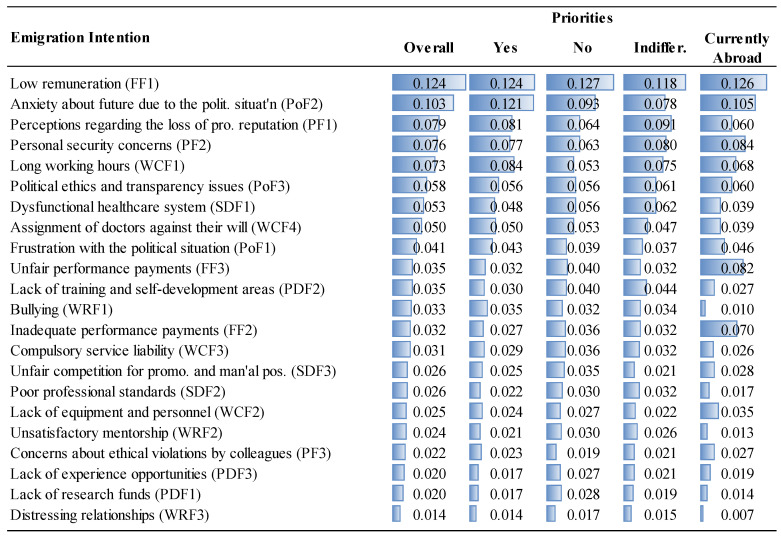
The priorities of factors (Overall vs. Emigration Intention).

The priorities of the factor groups and factors are given in Figure 6, Figure 7, Figure 8 and Figure 9 and Figure 10, Figure 11, Figure 12 and Figure 13, respectively. The analyses were also performed to observe the differences between participant groups in terms of their contextual variables such as age, gender, current position, and emigration intention. Note that for the emigration intention, the following classification was applied: people who want or definitely want to work/study abroad were put in the same group as those having the intention to emigrate; people who do not want or definitely do not want to work/study abroad were put in the same group as those having no intention to emigrate; and people who are currently abroad were examined separately.

Overall, it was found that political (0.203) and financial (0.191) factors as shown in Figure 6 and, more specifically, anxiety about their future, due to the political situation (PF2) and low remuneration (FF10), respectively, constitute the two most important factors driving the decision to emigrate for MDs in Turkey, as shown in Figure 10. On the other hand, working relationship (0.071) and personal development factors (0.075) were the least important factors (Figure 6). More specifically, as shown in Figure 10, the lowest priorities were distressing relationships (WRF3, 0.014), lack of research funds (PDF1, 0.020), and lack of experience opportunities (PDF3, 0.020). Considering the annual inflation rate of 72.31% in Turkey in 2022 as calculated by TUIK, a government agency [52], the importance attributed to the financial factors was highly expected. According to ENAG, a non-governmental inflation research group founded by independent academics, the inflation rate in 2022 was 137.55% [53]. The huge gap between the calculated inflation rate of these two agencies stems from the difference of items in the inflation basket. The consequences of the compulsory service requirement are well known and embraced by MDs, which explains its position at the bottom of the priority ranking.

Interestingly, political factors were far more important for specialty trainees (0.232) than for medical specialists (0.199) or for medical practitioners (0.198), as shown in Figure 6. Similarly, specialty trainees attributed significant importance to psychological factors (0.209) compared to other MDs. Although the low remuneration was the top priority (FF1, 0.121) for medical specialists, they underrated the unfair performance payment (FF3, 0.027), as shown in Figure 12. The results show that lack of training and self-development gain importance after MDs become specialists. This factor was not as important for specialty trainees as for other positions because they were already continuing their training. Unsatisfactory mentorship during medical or specialty training was far more important for specialty trainees, along with the other working condition factors.

While overall the participants highlighted the importance of political and financial factors in the emigration decision (in that order), women gave these factors less importance than men while giving greater importance to working conditions and psychological aspects than men (Figure 6).

When comparing the importance given to the political factors, the 26–33 age range as well as those older than 50 ranked the political factors as the most important overall reason to decide to emigrate, while ranking financial factors as the fourth most important factor after working conditions and psychological factors (Figure 7). Younger participants (18–25 age range) put less emphasis on political factors, which are among the highest ranked for other age groups. This is perhaps because their youth provides them with a longer period of time to expect changes in the current political situation. On the other hand, personal development factors, especially lack of research funds and training and self-development were more important for the 18–25 age range (Figure 11).

Observing the differences in terms of emigration intention, the importance of factors was slightly different for those participants with and those without the intention to emigrate. For example, MDs who are currently abroad overrated financial considerations (inadequate and unfair performance payments), while underrating the working relationship factors (Figure 13).

## 6. Conclusions and Further Suggestions

This study provides useful insights for policymakers to address the factors driving the decision to emigrate among MDs in Turkey and emphasizes, from a theoretical perspective, the significance of conducting a thorough contingent-decision analysis; that is, explicitly exploring generic and contextual factors. This approach provides critical details that policymakers can use to address the major reasons that cause an increasing number of physicians to emigrate from Turkey, and understanding these reasons will assist them to develop strategies to establish and to enhance sustainability in healthcare systems. However, most of the other identified decision factors are generalizable to other countries facing MD emigration problems. The contingent factors had 35.3% of the overall importance (see Figure 6, Figure 7, Figure 8, Figure 9, Figure 10, Figure 11, Figure 12 and Figure 13). Because of this, the present research shows the importance of conducting an explicit and thorough contingent-decision analysis to obtain important nuances which can be used to support policymakers’ management of the major issues that are leading to the loss of an increasing number of doctors.

According to the results of this study, MDs in Turkey are primarily motivated to emigrate by political and financial factors (Figure 6). Low salaries and concerns about the future political situation were identified as the main drivers of emigration. On the other hand, factors related to working relationships and personal development, such as compulsory service requirements, lack of research funding, and limited opportunities for experience, were less significant (Figure 10). While financial considerations were expected to be important since this has been found to be the case in many other countries (a generic factor), it was surprising that in Turkey the political considerations (contingent factors) were more critical for specialty trainees than for medical specialists or practitioners. Figure 11 shows that the contextual variable, anxiety about the future due to the political situation (PoF2), ranks second in importance to financial considerations and is particularly important for 26–41 year-olds, which is a valuable group of MDs to lose for a country. Also, those over 50 years old considered the anxiety factor as important as the previous younger segment of professionals (26–41 age range).

Interestingly, the study revealed that women considered political and financial factors to be less important than men, but gave more importance to working conditions and psychological aspects (Figure 6). This is consistent with the popular belief that women have a more focused concern for a good quality of life. On the other hand, personal development factors such as the lack of research funding and training opportunities were more important for the 18–25 age group, probably because this is the age range in which training is critical for a successful career. The findings that the emigration decision factors and in particular the contingent factors have different importance for MDs based on gender and age group suggest that the emigration problem should be addressed using a market-segmentation strategy.

For example, to alleviate the doctor emigration, policymakers could consider, among other incentives, increasing remuneration and taking measures to reassure the public situation, especially for specialty trainees who are more sensitive to political factors. Applying market segmentation, policymakers could focus on improving working conditions and psychological aspects, specifically for female doctors. In Turkey, only 11% of the chief physicians of public hospitals are females, based on 2021 data [54]. Ensuring gender equity in the work environment would lay the foundation and contribute to improving working conditions for female doctors. Also, training programs for administrators should be arranged to recognize and address the conscious or unconscious gender bias in the workplace.

Following this market-segmentation strategy, the present study suggests that providing opportunities for training and self-development could be effective in retaining younger doctors. Finally, policymakers could conduct a thorough analysis of the generic factors identified in this study to better understand the nuances of the emigration decision among MDs and develop effective policies accordingly.

The study’s results also suggest several potential areas for further research, such as investigating the actions and policies to mitigate MDs’ emigration in other nations, assessing the efficacy of policies aimed at addressing doctor shortages in Turkey and other countries, exploring the reasons for priority differences among various age groups in more detail, deciding what group segments the government should prioritize (e.g., young doctors, female, or a particular specialty), and examining the impact of working conditions and psychological factors on MD retention in Turkey, as well as with comparative studies with other countries. In effect, a contingent approach separates contextual factors (sourced through local experts) from generic factors (applicable to everybody), so the model can be applied to any other country through sensitivity analysis, changing, for example, the weight of the political or financial factors. This approach allows the use of the MD migration-decision model in different regional or national contexts.

## Figures and Tables

**Table 1:** Decision Model: Factors.

	ID	Factors	Sources
Psychological Factors (PFs)	PF1	Perceptions regarding the loss of professional reputation	[8]
	PF2	Personal security concerns (violence and harassment)	[3]
	PF3	Concerns about ethical violations by colleagues	Experts
Financial Factors (FFs)	FF1	Low remuneration	[3,4,5,6,7,8,23,24,25,26]
	FF2	Inadequate performance payments	[26]
	FF3	Unfair performance payments	Experts
Personal Development Factors (PDFs)	PDF1	Lack of research funds (research projects, conference participation, etc.)	Experts
	PDF2	Lack of training and self-development	[3,4,5,7,26]
	PDF3	Lack of experience opportunities	[5,25]
Political Factors (PoFs)	PoF1	Frustration with the political situation	[3,7]
	PoF2	Anxiety about their future	Experts
	PoF3	Political ethics and transparency issues	[3]
System Design Factors (SDFs)	SDF1	Dysfunctional healthcare system	[3]
	SDF2	Poor professional standards	[3]
	SDF3	Unfair competition for promotion and managerial positions	[3]
Working Relationship Factors (WRFs)	WRF1	Bullying	Experts
	WRF2	Unsatisfactory mentorship during medical or specialty training	[3]
	WRF3	Distressing relationships with peers or other healthcare staff	[3]
Working Conditions Factors (WCFs)	WCF1	Long working hours	[3,6,8,24]
	WCF2	Lack of preventive and therapeutic equipment and healthcare personnel	[3,4,24]
	WCF3	Compulsory service liability	Experts
	WCF4	Assignment of doctors against their will (inability of medical practitioners to choose the unit where they will work, etc.)	Experts

**Table 2:** Demographic information.

	Frequency	%
**Gender**		
Female	41	56.16%
Male	32	43.84%
**Age**		
18–25	3	4.11%
26–33	28	38.36%
34–41	15	20.55%
42–49	4	5.48%
≥50	23	31.51%
**Year of Graduation**		
Before 1980	1	1.37%
1980–1989	6	8.22%
1990–1999	17	23.29%
2000–2009	6	8.22%
2010–2019	36	49.32%
After 2020	7	9.59%
**Current Position**		
Specialty Trainee	9	12.33%
Medical Practitioner	33	45.21%
Medical Specialist	31	42.47%
**Emigration Intention**		
I definitely do not want to study/work abroad.	6	8.22%
I do not want to study/work abroad.	11	15.07%
I want to study/work abroad.	20	27.40%
I definitely want to study/work abroad.	16	21.92%
I haven’t decided about studying/working abroad yet.	17	23.29%
I am currently studying/working abroad.	3	4.11%

**Table 3:** The distribution of the area of specialty.

Area of Specialty	#	%
Family Medicine	4	10.00%
Gynecology and Obstetrics	4	10.00%
General Surgery	3	7.50%
Anesthesiology and Reanimation	2	5.00%
ENT (Ear, Nose, and Throat)	2	5.00%
Ophthalmology	2	5.00%
Pediatric Health and Diseases	2	5.00%
Psychiatry	2	5.00%
Cardiology	1	2.50%
Chest Diseases	1	2.50%
Emergency Medicine	1	2.50%
Endocrinology	1	2.50%
Forensic Medicine	1	2.50%
Hematology	1	2.50%
Infectious Diseases	1	2.50%
Internal Medicine	1	2.50%
Neurology	1	2.50%
Neurosurgery	1	2.50%
Orthopedics	1	2.50%
Pediatric Surgery	1	2.50%
Physical Therapy and Rehabilitation	1	2.50%
Plastic, Reconstructive and Aesthetic Surgery	1	2.50%
Public Health	1	2.50%
Thoracic Surgery	1	2.50%
Urology	1	2.50%
Not Available	2	5.00%
Total	40	100.00%

## Data Availability

The raw data supporting the conclusions of this article will be made available by the authors on request.

## Appendix A

Questionnaire
PART I
*Examining Factors Affecting Emigration Decision of MDs through the Analytic Hierarchy Process*

In the scope of this study, factors affecting the emigration decision of MDs in Turkey will be examined using a multiple criteria decision making method.

The survey is for academic purposes and does not impose any legal responsibility on the participants.

Your responses will be kept strictly confidential and never associated with your name. They will not be shared with third parties.

Your participation in this survey questionnaire is voluntary. You may choose not to participate. If you decide to participate in this research survey, you may withdraw at any time.

The questionnaire will take less than 15 min to complete.

Thank you very much for your time and cooperation.

Electronic Consent: Please select your choice below. Clicking on the “agree” button below indicates that you have read the above information and you voluntarily agree to participate. If you do not wish to participate in the survey, please decline participation by clicking on the “disagree” button.

- I agree to participate in the survey
- I disagree to participate in the survey

**Demographic Information**

1. Please select the option that best describes you:
  ○ I am a Medical Practitioner
  ○ I am a Medical Specialist
  ○ I am a Specialty Trainee
  ○ I am a Medical Student

*Those who select one of the first three options in the first question will be directed to the second question, and those who select the fourth option will be directed to the sixth question.*

*For medical practitioners, medical specialists, and specialty trainees:*

2. Please provide the medical school that you graduated from:
3. Year of graduation:
4. Area of specialty: (please indicate if you have not started your specialty training yet)
5. Please select the option that best describes you:
  ○ I am currently studying/working abroad.
  ○ I definitely want to study/work abroad.
  ○ I want to study/work abroad.
  ○ I do not want to study/work abroad.
  ○ I definitely do not want to study/work abroad.
  ○ I haven’t decided about studying/working abroad yet.

*For medical students:*

6. Please provide the medical school at which you are studying:
7. Please select the option that best describes you:
  ○ I am currently studying/working abroad.
  ○ I definitely want to study/work abroad.
  ○ I want to study/work abroad.
  ○ I do not want to study/work abroad.
  ○ I definitely do not want to study/work abroad.
  ○ I haven’t decided about studying/working abroad yet.

*Those who state that they are currently abroad in the fifth and seventh questions will be directed to the eighth question, and those who select the other options will be directed to the twelfth question.*

8. Which country are you in?
9. At what stage did you decide to study/work abroad?
  ○ Before medical training
  ○ During medical training
  ○ During specialty training
  ○ After specialty training
10. How many years have you been abroad?
11. Select the option that best describes your intention to return to the country:
  ○ Definitely will not return
  ○ Probably will not return
  ○ Indifferent
  ○ Probably will return
  ○ Definitely will return
12. Your age:
  ○ 18–25
  ○ 26–33
  ○ 34–41
  ○ 42–49
  ○ 50+
13. Your gender:
  ○ Female
  ○ Male
  ○ Prefer not to answer

**PART II: Pairwise Comparison Questions**

1. Of the affecting psychological factors, which one affects “emigration decision of MDs” more, and how much more?

1 = Equal 3 = Moderately more 5 = Strongly more 7 = Very strongly more 9 = Extremely more

Loss of Professional Reputation	9	8	7	6	5	4	3	2	1	2	3	4	5	6	7	8	9	Personal Security Concerns
Personal Security Concerns	9	8	7	6	5	4	3	2	1	2	3	4	5	6	7	8	9	Concerns About Ethical Violations by Colleagues
Concerns About Ethical Violations by Colleagues	9	8	7	6	5	4	3	2	1	2	3	4	5	6	7	8	9	Loss of Professional Reputation

2. Of the affecting financial factors, which one affects “emigration decision of MDs” more, and how much more?

1 = Equal 3 = Moderately more 5 = Strongly more 7 = Very strongly more 9 = Extremely more

Low Remuneration	9	8	7	6	5	4	3	2	1	2	3	4	5	6	7	8	9	Inadequate Performance Payments
Inadequate Performance Payments	9	8	7	6	5	4	3	2	1	2	3	4	5	6	7	8	9	Unfair Performance Payments
Unfair Performance Payments	9	8	7	6	5	4	3	2	1	2	3	4	5	6	7	8	9	Low Remuneration

3. Of the affecting personal development factors, which one affects “emigration decision of MDs” more, and how much more?

1 = Equal 3 = Moderately more 5 = Strongly more 7 = Very strongly more 9 = Extremely more

Lack of Research Funds	9	8	7	6	5	4	3	2	1	2	3	4	5	6	7	8	9	Lack of Training and Self-development Areas
Lack of Training and Self-development Areas	9	8	7	6	5	4	3	2	1	2	3	4	5	6	7	8	9	Lack of Experience Opportunities
Lack of Experience Opportunities	9	8	7	6	5	4	3	2	1	2	3	4	5	6	7	8	9	Lack of Research Funds

4. Of the affecting political factors, which one affects “emigration decision of MDs” more and how much more?

1 = Equal 3 = Moderately more 5 = Strongly more 7 = Very strongly more 9 = Extremely more

Frustration with the Political Situation	9	8	7	6	5	4	3	2	1	2	3	4	5	6	7	8	9	Anxiety About Their Future due to the Political Situation
Anxiety About Their Future due to the Political Situation	9	8	7	6	5	4	3	2	1	2	3	4	5	6	7	8	9	Political Ethics and Transparency Issues
Political Ethics and Transparency Issues	9	8	7	6	5	4	3	2	1	2	3	4	5	6	7	8	9	Frustration with the Political Situation

5. Of the affecting system design factors, which one affects “emigration decision of MDs” more, and how much more?

1 = Equal 3 = Moderately more 5 = Strongly more 7 = Very strongly more 9 = Extremely more

Dysfunctional Healthcare System	9	8	7	6	5	4	3	2	1	2	3	4	5	6	7	8	9	Poor Professional Standards
Poor Professional Standards	9	8	7	6	5	4	3	2	1	2	3	4	5	6	7	8	9	Unfair Competition for Promotion and Managerial Positions
Unfair Competition for Promotion and Managerial Positions	9	8	7	6	5	4	3	2	1	2	3	4	5	6	7	8	9	Dysfunctional Healthcare System

6. Of the affecting working relationship factors, which one affects “emigration decision of MDs” more, and how much more?

1 = Equal 3 = Moderately more 5 = Strongly more 7 = Very strongly more 9 = Extremely more

Bullying	9	8	7	6	5	4	3	2	1	2	3	4	5	6	7	8	9	Unsatisfactory Mentorship
Unsatisfactory Mentorship	9	8	7	6	5	4	3	2	1	2	3	4	5	6	7	8	9	Distressing Relationships with Peers or Other Healthcare Staff
Distressing Relationships with Peers or Other Healthcare Staff	9	8	7	6	5	4	3	2	1	2	3	4	5	6	7	8	9	Bullying

7. Of the affecting working conditions factors, which one affects “emigration decision of MDs” more, and how much more?

1 = Equal 3 = Moderately more 5 = Strongly more 7 = Very strongly more 9 = Extremely more

Long Working Hours	9	8	7	6	5	4	3	2	1	2	3	4	5	6	7	8	9	Lack of Preventive and Therapeutic Equipment and Healthcare Personnel
Lack of Preventive and Therapeutic Equipment and Healthcare Personnel	9	8	7	6	5	4	3	2	1	2	3	4	5	6	7	8	9	Compulsory Service Liability
Compulsory Service Liability	9	8	7	6	5	4	3	2	1	2	3	4	5	6	7	8	9	Assignment of Doctors Against Their Will
Long Working Hours	9	8	7	6	5	4	3	2	1	2	3	4	5	6	7	8	9	Compulsory Service Liability
Lack of Preventive and Therapeutic Equipment and Healthcare Personnel	9	8	7	6	5	4	3	2	1	2	3	4	5	6	7	8	9	Assignment of Doctors Against Their Will
Assignment of Doctors Against Their Will	9	8	7	6	5	4	3	2	1	2	3	4	5	6	7	8	9	Long Working Hours

8. Of the affecting factors, which one affects “emigration decision of MDs” more, and how much more?

1 = Equal 3 = Moderately more 5 = Strongly more 7 = Very strongly more 9 = Extremely more

Psychological Factors	9	8	7	6	5	4	3	2	1	2	3	4	5	6	7	8	9	Financial Factors
Financial Factors	9	8	7	6	5	4	3	2	1	2	3	4	5	6	7	8	9	Personal Development Factors
Personal Development Factors	9	8	7	6	5	4	3	2	1	2	3	4	5	6	7	8	9	Political Factors
Political Factors	9	8	7	6	5	4	3	2	1	2	3	4	5	6	7	8	9	System Design Factors
System Design Factors	9	8	7	6	5	4	3	2	1	2	3	4	5	6	7	8	9	Working Relationship Factors
Working Relationship Factors	9	8	7	6	5	4	3	2	1	2	3	4	5	6	7	8	9	Working Conditions Factors
Psychological Factors	9	8	7	6	5	4	3	2	1	2	3	4	5	6	7	8	9	Personal Development Factors
Financial Factors	9	8	7	6	5	4	3	2	1	2	3	4	5	6	7	8	9	Political Factors
Personal Development Factors	9	8	7	6	5	4	3	2	1	2	3	4	5	6	7	8	9	System Design Factors
Political Factors	9	8	7	6	5	4	3	2	1	2	3	4	5	6	7	8	9	Working Relationship Factors
System Design Factors	9	8	7	6	5	4	3	2	1	2	3	4	5	6	7	8	9	Working Conditions Factors
Psychological Factors	9	8	7	6	5	4	3	2	1	2	3	4	5	6	7	8	9	Political Factors
Financial Factors	9	8	7	6	5	4	3	2	1	2	3	4	5	6	7	8	9	System Design Factors
Personal Development Factors	9	8	7	6	5	4	3	2	1	2	3	4	5	6	7	8	9	Working Relationship Factors
Political Factors	9	8	7	6	5	4	3	2	1	2	3	4	5	6	7	8	9	Working Conditions Factors
Psychological Factors	9	8	7	6	5	4	3	2	1	2	3	4	5	6	7	8	9	System Design Factors
Financial Factors	9	8	7	6	5	4	3	2	1	2	3	4	5	6	7	8	9	Working Relationship Factors
Personal Development Factors	9	8	7	6	5	4	3	2	1	2	3	4	5	6	7	8	9	Working Conditions Factors
Psychological Factors	9	8	7	6	5	4	3	2	1	2	3	4	5	6	7	8	9	Working Relationship Factors
Financial Factors	9	8	7	6	5	4	3	2	1	2	3	4	5	6	7	8	9	Working Conditions Factors
Working Conditions Factors	9	8	7	6	5	4	3	2	1	2	3	4	5	6	7	8	9	Psychological Factors

## Appendix B

**Table A1:** Inconsistencies (Overall).

Pairwise Comparisons (wrt.)	Overall	Current Position			Gender		Emigration Intention				Age				
		Medical Specialist	Specialty Trainee	Pract.	Women	Men	Yes	No	Indiff.	Abroad	18–25	26–33	34–41	42–49	>50
Goal	0.44%	0.68%	2.58%	0.62%	0.84%	0.55%	1.03%	0.55%	0.65%	4.13%	9.46%	0.62%	1.12%	3.33%	0.56%
Psychological Factors	0.46%	1.02%	1.14%	0.08%	0.19%	0.75%	0.29%	3.11%	0.00%	0.10%	6.52%	0.09%	3.85%	0.01%	0.10%
Financial Factors	0.04%	0.43%	7.26%	0.27%	0.07%	0.02%	1.19%	1.41%	5.31%	4.49%	0.02%	0.12%	6.53%	2.84%	0.08%
Personal Dev. Factors	0.01%	1.19%	2.38%	0.19%	0.43%	0.45%	0.30%	2.32%	0.00%	0.07%	0.22%	0.01%	0.11%	3.29%	1.85%
Political Factors	0.34%	0.02%	0.14%	2.23%	1.60%	0.00%	0.02%	0.87%	4.22%	0.83%	0.68%	0.08%	4.18%	0.27%	0.14%
System Design Factors	0.03%	0.96%	0.21%	0.42%	1.45%	1.62%	0.63%	0.01%	1.36%	1.49%	0.00%	0.02%	0.27%	6.72%	0.78%
Working Rel. Factors	0.57%	0.66%	0.03%	0.78%	0.03%	2.21%	0.95%	0.01%	1.49%	0.78%	4.54%	1.43%	0.61%	1.64%	0.62%
Working Cond. Factors	0.26%	0.89%	0.25%	0.23%	0.43%	0.25%	0.41%	0.03%	0.93%	2.32%	5.66%	0.23%	1.50%	4.86%	0.44%

## References

[1] Byrne J.-P., Conway E., McDermott A.M., Matthews A., Prihodova L., Costello R.W., Humphries N. (2021). How the Organisation of Medical Work Shapes the Everyday Work Experiences Underpinning Doctor Migration Trends: The Case of Irish-Trained Emigrant Doctors in Australia. Health Policy.

[2] Mehra R., Sharma M.K. (2021). Measures of Sustainability in Healthcare. Sustain. Anal. Model..

[3] Akl E.A., Maroun N., Major S., Afif C., Chahoud B., Choucair J., Sakr M., Schünemann H.J. (2007). Why Are You Draining Your Brain? Factors Underlying Decisions of Graduating Lebanese Medical Students to Migrate. Soc. Sci. Med..

[4] Botezat A., Moraru A. (2020). Brain Drain from Romania: What Do We Know so Far about the Romanian Medical Diaspora?. East. J. Eur. Stud..

[5] Gouda P., Kitt K., Evans D.S., Goggin D., McGrath D., Last J., Hennessy M., Arnett R., O’Flynn S., Dunne F. (2015). Ireland’s Medical Brain Drain: Migration Intentions of Irish Medical Students. Hum. Resour. Health.

[6] Humphries N., McDermott A.M., Conway E., Byrne J.P., Prihodova L., Costello R., Matthews A. (2019). ‘Everything Was Just Getting Worse and Worse’: Deteriorating Job Quality as a Driver of Doctor Emigration from Ireland. Hum. Resour. Health.

[7] Onah C.K., Azuogu B.N., Ochie C.N., Akpa C.O., Okeke K.C., Okpunwa A.O., Bello H.M., Ugwu G.O. (2022). Physician Emigration from Nigeria and the Associated Factors: The Implications to Safeguarding the Nigeria Health System. Hum. Resour. Health.

[8] Sheikh A., Naqvi S.H.A., Sheikh K., Naqvi S.H.S., Bandukda M.Y. (2012). Physician Migration at Its Roots: A Study on the Factors Contributing towards a Career Choice Abroad among Students at a Medical School in Pakistan. Glob. Health.

[9] Genc K. (2022). Turkish Doctors Emigrate amid Low Pay and Rising Violence. Lancet.

[10] Türk Tabipleri Birliği @ttborgtr TTB’ye “İyi Hal Belgesi” Başvuru Sayısı; Aralık Ayında 268, 2022 Yılında 2685 oldu. Image attached. Twitter 2023. https://twitter.com/ttborgtr/status/1609828230948683782?lang=en.

[11] Health Resources—Doctors—OECD Data. http://data.oecd.org/healthres/doctors.htm.

[12] Demïroğlu T., Kilinç E., Atay E. (2015). Violence towards Health Professionals: The Case of Kilis City. J. Health Sci..

[13] Devebakan N. (2018). Workplace Violence in Health Care Organizations and Evaluation of “Code White” Application in Dokuz Eylul University Application and Research Hospital. Suleyman Demirel Univ. J. Fac. Econ. Adm. Sci..

[14] Mïlet M., Yanik A. (2017). Workplace Violence against Health Workers. Int. J. Health Manag. Strateg. Res..

[15] The Central Bank of Turkey. https://www.tcmb.gov.tr/wps/wcm/connect/EN/TCMB+EN/Main+Menu/Statistics/Exchange+Rates/Indicative+Exchange+Rates.

[16] Türk-IS Temmuz 2022. Açlik Ve Yoksulluk Siniri. https://www.turkis.org.tr/temmuz-aclik-yoksulluk-siniri/.

[17] Turkish Medical Association Sağlık Bakanlığı Yeni Ek Ödeme Yönetmeliği: Kâr Odaklı ve Geçici Düzenlemelere Değil, Toplumu Önceleyen Yeni Bir Sağlık Sistemine İhtiyaç Vardır. https://www.ttb.org.tr/haber_goster.php?Guid=c018f720-1d62-11ed-88fa-ab1256785955.

[18] Mu E., Cooper O., Topcu Y.I., Onsel Ekici S., Kabak O., Aktas E., Ozaydin O. (2022). A Contingency Approach to Multi-Criteria Decision-Making: A Search for Validity through Rigor and Relevance. New Perspectives in Operations Research and Management Science: Essays in Honor of Fusun Ulengin.

[19] Nutt P.C., Wilson D.C. (2010). Crucial Trends and Issues in Strategic Decision Making. Handbook of Decision Making.

[20] Mintzberg H., Raisinghani D., Theoret A. (1976). The Structure of” Unstructured” Decision Processes. Adm. Sci. Q..

[21] Carling J., Collins F. (2018). Aspiration, Desire and Drivers of Migration. J. Ethn. Migr. Stud..

[22] Van Hear N., Bakewell O., Long K. (2018). Push-Pull plus: Reconsidering the Drivers of Migration. J. Ethn. Migr. Stud..

[23] Domagała A., Kulbat A., Parzonka K. (2022). Emigration from the Perspective of Polish Health Professionals–Insights from a Qualitative Study. Front. Public Health.

[24] Lawal L., Lawal A.O., Amosu O.P., Muhammad-Olodo A.O., Abdulrasheed N., Abdullah K.-R., Kuza P.B., Aborode A.T., Adebisi Y.A., Kareem A.A. (2022). The COVID-19 Pandemic and Health Workforce Brain Drain in Nigeria. Int. J. Equity Health.

[25] Stilwell B., Diallo K., Zurn P., Vujicic M., Adams O., Dal Poz M. (2004). Migration of Health-Care Workers from Developing Countries: Strategic Approaches to Its Management. Bull. World Health Organ..

[26] Diallo K. (2004). Data on the Migration of Health-Care Workers: Sources, Uses, and Challenges. Bull. World Health Organ..

[27] Mu E., Stern H. (2018). A Contingent/Assimilation Framework for Public Interorganizational Systems Decisions: Should the City of Pittsburgh and Allegheny County Consolidate Information Technology Services?. Int. J. Inf. Technol. Decis. Mak..

[28] Saaty T.L. (1980). Decision Making for Leaders: The Analytical Hierarchy Process.

[29] Ishizaka A., Mu E. (2023). What Is so Special about the Analytic Hierarchy and Network Process?. Ann. Oper. Res..

[30] Saaty T.L. (2000). Fundamentals of Decision Making and Priority Theory with the Analytic Hierarchy Process.

[31] Ishizaka A., Nemery P. (2013). Multi-Criteria Decision Analysis: Methods and Software.

[32] Saaty T.L. (1996). Decision Making with Dependence and Feedback: The Analytic Network Process.

[33] Kabbash I., El-Sallamy R., Zayed H., Alkhyate I., Omar A., Abdo S. (2021). The Brain Drain: Why Medical Students and Young Physicians Want to Leave Egypt. East. Mediterr. Health J..

[34] Turkey Ministry of Health (2018). 2017 Annual Report.

[35] Turkey Ministry of Health (2022). 2021 Annual Report.

[36] Shi L., Li G., Hao J., Wang W., Chen W., Liu S., Yu Z., Shi Y., Ma Y., Fan L. (2020). Psychological Depletion in Physicians and Nurses Exposed to Workplace Violence: A Cross-Sectional Study Using Propensity Score Analysis. Int. J. Nurs. Stud..

[37] International Labour Office ILO (2002). Framework Guidelines for Addressing Workplace Violence in the Health Sector.

[38] The Centre for Assessment, Selection and Placement. https://www.osym.gov.tr/TR,23885/2022-yuksekogretim-kurumlari-sinavi-yks-yuksekogretim-programlari-ve-kontenjanlari-kilavuzu.html.

[39] Turkey Ministry of Health (2022). The Ministry of Health Payment Regulation.

[40] The Presidency of the Republic of Turkey, Legislation Information System. https://www.mevzuat.gov.tr/mevzuat?MevzuatNo=3359&MevzuatTur=1&MevzuatTertip=5.

[41] Zoho Survey. https://www.zoho.com/survey/index.html.

[42] Saaty T.L., Peniwati K. (2007). Group Decision Making: Drawing out and Reconciling Differences.

[43] Migration and Home Affairs. https://home-affairs.ec.europa.eu/networks/european-migration-network-emn/emn-asylum-and-migration-glossary/glossary/emigration_en.

[44] Carling J. (2002). Migration in the Age of Involuntary Immobility: Theoretical Reflections and Cape Verdean Experiences. J. Ethn. Migr. Stud..

[45] Carling J., Schewel K. (2018). Revisiting Aspiration and Ability in International Migration. J. Ethn. Migr. Stud..

[46] Incekas A.B., Kadaifci C., Calisir F. (2022). Brain Drain: A Multi-Criteria Decision Model. Proceedings of the Industrial Engineering in the Internet-of-Things World: Selected Papers from the Virtual Global Joint Conference on Industrial Engineering and Its Application Areas, GJCIE 2020.

[47] Al Husein N., Wagner N. (2023). Determinants of Intended Return Migration among Refugees: A Comparison of Syrian Refugees in Germany and Turkey. Int. Migr. Rev..

[48] Mensah J., Owusu Ansah A. (2022). Reflections on Return Migration: Understanding How African Immigrants in Canada Contemplate Return. Int. Migr..

[49] De Haas H., Fokkema T., Fihri M.F. (2015). Return Migration as Failure or Success? The Determinants of Return Migration Intentions among Moroccan Migrants in Europe. J. Int. Migr. Integr..

[50] Super Decisions Software. https://www.superdecisions.com/.

[51] Creative Decisions Foundation. https://www.creativedecisions.net/resources/.

[52] Tüketici Fiyat Endeksi, Aralık 2022. https://data.tuik.gov.tr/Bulten/Index?p=Tuketici-Fiyat-Endeksi-Aralik-2022-49651.

[53] ENAGRUP—Enflasyon Araştırma Grubu. https://enagrup.org/.

[54] Kaya R.R., Alkan A. (2021). A Study on The Proile of Physicians Who Work in Administrative Positions in the Public Hospitals in Turkey. Manisa Celal Bayar Univ. J. Inst. Health Sci..

